# Implementation of healthy food environment policies to prevent nutrition-related non-communicable diseases in Ghana: National experts’ assessment of government action

**DOI:** 10.1016/j.foodpol.2020.101907

**Published:** 2020-05

**Authors:** Amos Laar, Amy Barnes, Richmond Aryeetey, Akua Tandoh, Kristin Bash, Kobby Mensah, Francis Zotor, Stefanie Vandevijvere, Michelle Holdsworth

**Affiliations:** a Department of Population, Family and Reproductive Health, School of Public Health, University of Ghana, Accra, Ghana; b Public Health Section, School of Health and Related Research, University of Sheffield, Sheffield, UK; c Department of Marketing and Entrepreneurship, University of Ghana Business School, Accra, Ghana; d Department of Family and Community Health, School of Public health, University of Health and Allied Sciences Ho, Ghana; e School of Population Health, The University of Auckland, New Zealand; f UMR NUTRIPASS, French National Research Institute for Sustainable Development- IRD, Montpellier, France

**Keywords:** Healthy food environment, Policy implementation, Obesity, Nutrition-related non-communicable diseases, Policy, Lower-middle Income country

## Abstract

Nutrition-related non-communicable diseases (NR-NCDs) are a global health problem, increasingly recognised as driven by unhealthy food environments. Yet little is known about government action to implement food environment-relevant policies, particularly in low-and lower-middle income countries. This study assessed government action, implementation gaps, and priorities to improve the food environment in Ghana. Using the Healthy Food-Environment Policy Index (Food-EPI), a panel comprising government and independent experts (n = 19) rated government action to improve the healthiness of food environment in Ghana against international best practices and according to steps within a policy cycle. Forty-three good practice indicators of food environment policy and infrastructure support were used, with ratings informed by systematically collected evidence of action validated by government officials. Following the rating exercise, the expert panel proposed and prioritized actions for government implementation. Three-quarters of all good practice indicators were rated at ‘low’/‘very little’ implementation. Restricting the marketing of breast milk substitutes was the only indicator rated “very high”. Of ten policy actions prioritized for implementation, restricting unhealthy food marketing in children’s settings and in the media were ranked the highest priority. Providing sufficient funds for nationally-relevant research on nutrition and NCDs was the highest priority infrastructure-support action. Other priority infrastructure-support actions related to leadership, monitoring and evaluation. This study identified gaps in Ghana’s implementation of internationally-recommended policies to promote healthy food environments. National stakeholders recommended actions, which will require legislation and leadership. The findings provide a baseline for measuring government progress towards implementing effective policies to prevent NR-NCDs.

## Highlights

• There is insufficient implementation of food environment policies in Ghana.
• Government action to restrict marketing of breastmilk substitutes is advanced in Ghana.
• Legislation to regulate advertising/sale of unhealthy food is an identified priority.
• Leadership, monitoring and evaluation are needed to support policy infrastructure.
• Funding for nationally relevant research on NCDs is a high infrastructure priority.

## 1 Introduction

Reducing obesity and other nutrition-related non-communicable diseases (NR-NCDs) remains an urgent task worldwide. Globally, the prevalence of obesity has soared since 1975 (Abarca-Gómez et al., 2017). In 2016, more than 1.9 billion adults were estimated to be overweight (greater than650 million of whom were obese) and over 340 million children and adolescents aged 5–19 years overweight or obese (Islam et al., 2014). Increasingly, populations are exposed to unhealthy food environments that influence the risk of obesity and other NR-NCDs (including type 2 diabetes, cardiovascular diseases and many cancers) (Lachat et al., 2013, Cairns et al., 2013). To address this problem, and achieve the World Health Organisation’s (WHO) target to halt the rise in global diabetes and obesity prevalence by 2025 (World Health Organization, 2014) comprehensive action needs to be taken by governments, as well as the food industry. To make progress in controlling dietary risk factors associated with NR-NCDs, a thorough understanding of the status and progress in implementing food environment relevant policies, regulations, and programs by national governments is required, (Swinburn et al., 2013, World Health Organization. Noncommunicable Diseases Progress Monitor 201Available at: https://www.who.int/nmh/publications/ncd-progress-monitor-, 2017) given that these are increasingly recognized as a crucial entry point to tackle unhealthy diets (United Nations, 2018.). In public health, the food environment is defined as “the collective physical, economic, policy and sociocultural surroundings, opportunities and conditions that influence people’s food and beverage choices and nutritional status” (Swinburn et al., 2013).

Tackling unhealthy diets and unhealthy food environments is a policy challenge in LMICs and in Sub-Saharan Africa (SSA) in particular; in part because of the concerning rate at which obesity and other NR-NCDs are increasing. The 2015 Global Burden of Disease Report (Institute of Health Metrics and Evaluation (IHME), 2015) highlighted, for example, a 1400% increase in rates of adult obesity in Burkina Faso and over 500% increase in Ghana, Togo, Ethiopia and Benin between 1980 and 2015. The Report further indicated that 8 out of the 20 countries with the fastest-rising rates of adult obesity are in Africa. (Institute of Health Metrics and Evaluation (IHME), 2015) Globally, there is evidence that such increases in obesity rates are due to unhealthy food environments and food system failures (Abarca-Gómez et al., 2017, Freudenberg, 2014, Kessler, 2010). In Ghana, such food system failures have contributed to a changing quality of diets and the rise in obesity and NR-NCDs (Ofori-Asenso et al., 2016). Ghana is at an advanced stage of the nutrition transition, experiencing rapid urbanization, and increasing overweight/obesity and related NCDs (Ghana Statistical Service (GSS) GHSG, and ICF International, 2014). Overall, overweight/obesity among women in fertility age has increased from 10% in 1993 to 40% in 2014 (Ghana Statistical Service (GSS) GHSG, and ICF International. Ghana Demographic and Health Survey, 2014, Ghana Statistical Service - GSS and Macro International. Ghana Demographic and Health Survey, 1993). Having traditionally focused on addressing communicable diseases and maternal and child health, efforts by the Ghana health delivery system to address NCDs (Ministry of Health [MOH-Ghana], 2012) are not only nascent, but scant, and not effectively coordinated (Laar et al., 2019). Although some studies and grey literature on existing policies and approaches on obesity and NR-NCD prevention in Africa exist (for example, the WHO NCD Progress Monitor (World Health Organization, 2017), which includes Ghana), none has assessed or ranked government action in Ghana or West Africa in relation to creating healthy food environments.

The world over, healthiness of food environments is a key driver of the nutritional quality of population diets, but food environments are not regularly monitored and implementation of most policies tackling those food environments is slow and inadequate. In response, the International Network for Food and Obesity/Non-communicable Diseases Research, Monitoring and Action Support (INFORMAS), was set up to monitor and benchmark food environments and policies internationally. Among others, INFORMAS seeks to increase the accountability of governments and the food industry for action to reduce diet-related NCDs and their associated inequalities. In 2013, the Network developed the Healthy Food Environment Policy Index (Food‐EPI) (Swinburn et al., 2013) to support governments in adopting policies to improve the food environment. The Food-EPI is an international standardized tool which comprises more than 40 ‘good practice’ indicators across seven food environment policy domains (including: food composition, nutrition labelling, food promotion, food prices, food provision) and six infrastructure support domains (including leadership, multi-sectoral action) that influence food environments (Swinburn et al., 2013). The Food-EPI tool and associated process can help identify critical gaps in national policy action, and support the identification and prioritization of actions to address them, by comparing national performance with international best practices (Swinburn et al., 2013, Vandevijvere and Swinburn, 2014). Although the Food-EPI was developed for implementation in countries globally and not just for use in High Income Countries (HICs), the tool has so far been largely implemented in HICs. Globally – as of 2018 - the Food-EPI tool had been successfully implemented in 11 countries: in 6 HICs and 5 Upper Middle Income Countries (UICs); none in Lower-Middle Income Countries (LMICs) and Low income countries (LICs).

This paper reports on the application of the Food-EPI tool in Ghana, West Africa, between October 2017 and January 2019, as part of the Dietary Transitions in Ghanaian Cities Project (hereafter referred to as DFC Project). We explain how we worked with an expert panel to assess the extent of government implementation of recommended food environment policies compared to international best practice and in relation to steps within a policy cycle. We also report on how the Food-EPI exercise supported the identification and prioritization of actions for implementation by the Ghanaian government. We emphasize the importance of this process in monitoring the implementation of internationally recommended food environment policies, as a critical part of ensuring progress towards better nutritional health.

Our study is one of the first to successfully apply the Food-EPI process to appraise government actions on NR-NCDs and prioritize actions for implementation in a LMIC setting. Our other effort applying the Food EPI process in Africa was in Kenya (Asiki et al., 2020). The paper discusses how specific policy interventions, such as fiscal, regulatory, and provisioning measures provide a crucial entry point for improving the food environment and contribute to controlling NR-NCDs in Ghana. This work supports current calls to improve food environments in LMICs, and goes further to demonstrate that it is possible to deploy the INFORMAS’ Food-EPI in Africa and other LMICs.'

## 2 Materials and methods

### 2.1 The Food-EPI process

**Fig. 1:**
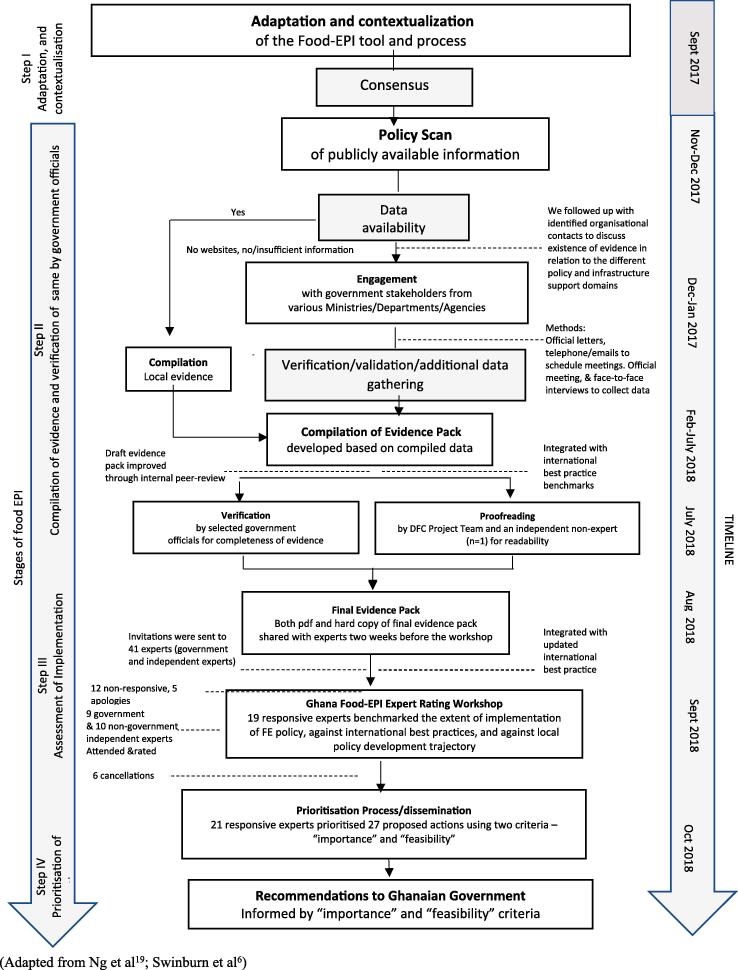
The Food-EPI process in Ghana, 2017–2018

There were four main steps in the Food-EPI process in Ghana, which are summarized below (Fig. 1).

#### 2.1.1 Step I. Tool and process adaptation

The Food-EPI tool was tailored to the Ghanaian context by the research team, in consultation with the authors of the original Food-EPI protocol (Swinburn et al., 2013), during a workshop held in Accra in September 2017. The workshop was facilitated by co-author SV. Minor modifications were made to the original 47-indicator tool: 2 new indicators were introduced to ascertain whether effective policies had been implemented by government to restrict the marketing of breast milk substitutes and assess if food hygiene policies are robust and being enforced, where needed, by national and local government; 4 existing indicators associated with Food Provision, Food in Retail, Platforms for Interaction and Health in all Policies were either deleted or merged. A few others were rephrased to enhance clarity. The final Ghana Food-EPI tool comprised 13 policy and infrastructure support domains and 44 indicators (see Table 1). Forty-three of these 44 indicators were used in the rating workshop; one indicator on food hygiene, for which no international best practice examples exist, was not used.

**Table 1:** Policy and infrastructure-support indicators of the Ghana Food-EPI tool.

*Policy Domain Indicators*
**Food Composition**
**COMP 1:** Food composition targets/standards have been established for *processed foods* by the government for the content of the nutrients of concern in certain foods or food groups if they are major contributors to population intakes of these nutrients of concern (trans fats and added sugars in processed foods, salt in bread salt in snacks etc.))
**COMP 2:** Food composition targets/standards have been established by the government *for out-of-home meals in food service outlets* (such as fast food joints, food kiosks, check-check joints, restaurants, and other local food vendors) for the content of the nutrients of concern in certain foods or food groups if they are major contributors to population intakes of these nutrients of concern (e.g. trans fats, added sugars, salt, saturated fat, saturated fat in commercial frying fats/oils)
**Food Labelling**
**LABEL1**: Ingredient lists and nutrient declarations in line with Codex recommendations are present on the labels of packaged foods
**LABEL2:** Robust, evidence-based regulatory systems are in place for approving/reviewing claims on foods, so that consumers are protected against unsubstantiated and misleading nutrition and health claims
**LABEL3:** A single, consistent, interpretive, evidence-informed front-of-pack supplementary nutrition information system, which readily allows consumers to assess a product’s healthiness, is applied to packaged foods
**LABEL4:** A consistent, single, simple, clearly-visible system of labelling the menu boards of quick service restaurants (i.e. fast food chains) is applied by the government, which allows consumers to interpret the nutrient quality and energy content of foods and meals on sale
**Food Promotion**
**PROMO1:** Effective policies are implemented by the government to restrict exposure and power of promotion of unhealthy foods to or for children through *broadcast media (TV, radio)*
**PROMO2:** Effective policies are implemented by the government to restrict exposure and power of promotion of unhealthy foods to or for children *through non-broadcast media* (e.g. Internet, social media, food packaging, sponsorship, religious events, outdoor advertising including around schools)
**PROMO3:** Effective policies are implemented by the government to ensure that unhealthy foods are not commercially promoted to or for children *in settings where children gather* (e.g. preschools, schools, sport and cultural events)
**PROMO4:** Effective policies are implemented by the government to restrict the marketing of breastmilk substitutes
**PRICES1:** Taxes or levies on healthy foods are minimised to encourage healthy food choices where possible (e.g. low or no sales tax, excise, value-added or import duties on fruit and vegetables)
**Food Prices**
**PRICES2:** Taxes or levies on unhealthy foods (e.g. sugar-sweetened beverages, foods high in nutrients of concern) are in place and increase the retail prices of these foods by at least 10% to discourage unhealthy food choices where possible, and these taxes are reinvested to improve population health
**PRICES3:** The intent of existing subsidies on foods, including infrastructure funding support (e.g. research and development, supporting markets or transport systems), is to favour healthy rather than unhealthy foods
**PRICES4:** The government ensures that food-related income support programs are for healthy foods
**Food Provision**
**PROV1:** The government ensures that there are clear, consistent policies (including nutrition standards) implemented *in schools and early childhood education services* for food service activities (canteens, food at events, fundraising, promotions, vending machines etc.) to provide and promote healthy food choices
**PROV2:** The government ensures that there are clear, consistent policies *in other public sector settings* for food service activities (canteens, hospitals, clinics, food at events, fundraising, promotions, vending machines, public procurement standards etc.) to provide and promote healthy food choices.
**PROV3:** The Government ensures that there are good support and training systems to help schools and other public sector organisations and their caterers meet the healthy food service policies and guidelines
**Food in Retail**
**RETAIL1:** Zoning laws and policies are robust enough and are being used, where needed, by local governments to place limits on the density or placement of quick serve restaurants or other outlets selling mainly unhealthy foods in communities, and to encourage the availability of outlets selling healthy options such as fresh fruit and vegetables
**RETAIL2:** The Government ensures existing support systems are in place to encourage food stores and food service outlets to promote the availability of healthy foods and to limit the promotion and availability of unhealthy foods
**RETAIL 3:** Food hygiene policies are robust enough and are being enforced, where needed, by national and local government to protect human health and consumers’ interests in relation to food.
**Food Trade and Investment**
**TRADE1:** The Government undertakes risk impact assessments before and during the negotiation of trade and investment agreements, to identify, evaluate and minimize the direct and indirect negative impacts of such agreements on population nutrition and health
**TRADE2:** The government adopts measures to manage investment and protect their regulatory capacity with respect to public health nutrition
***Infrastructure Support Domain Indicators***
**Leadership**
**LEAD1:** There is strong, visible, political support (at the Head of Government / Cabinet level) for improving food environments, population nutrition, diet-related NCDs and their related inequalities
**LEAD2:** Clear population intake targets have been established by the government for the nutrients of concern to meet WHO and national recommended dietary intake levels
**LEAD3:** Clear, interpretive, evidence-informed food-based dietary guidelines have been established and implemented
**LEAD4:** There is a comprehensive, transparent, up-to-date and government-owned implementation plan, - linked to national needs and priorities -to improve food environments, reduce the intake of the nutrients of concern to meet WHO and national recommended dietary intake levels, and reduce diet-related NCDs
**LEAD5:** Government priorities have been established to reduce inequalities or protect vulnerable populations in relation to diet, nutrition, obesity and NCDs
**Governance**
**GOVER1:** There are robust procedures to restrict commercial influences on the development of policies related to food environments where they have conflicts of interest with improving population nutrition
**GOVER2:** Policies and procedures are implemented for using evidence in the development of food policies
**GOVER3:** Policies and procedures are implemented for ensuring transparency in the development of food policies
**GOVER4:** The government ensures access to comprehensive nutrition information and key documents (e.g. budget documents, annual performance reviews and health indicators) for the public
**Monitoring and Evaluation**
**MONIT1:** Monitoring systems, implemented by the government, are in place to regularly monitor food environments (especially for food composition for nutrients of concern, food promotion to children, and nutritional quality of food in schools and other public-sector settings), against codes/guidelines/standards/targets.
**MONIT2:** There is regular monitoring of adult and childhood nutrition status and population intakes against specified intake targets or recommended daily intake levels.
**MONIT3**: There is regular monitoring of adult and childhood overweight and obesity prevalence using anthropometric measurements
**MONIT4:** There is regular monitoring of the prevalence of NCD risk factors and occurrence rates (e.g. prevalence, incidence, mortality) for the main diet-related NCDs
**MONIT5:** There is sufficient evaluation of major programs and policies to assess effectiveness and contribution to achieving the goals of the nutrition and health plans
**MONIT6:** Progress towards reducing health inequalities or health impacts in vulnerable populations and societal and economic determinants of health are regularly monitored
**Funding**
**FUND1:** Funding for the promotion of healthy eating and healthy food environments, as a proportion of total health spending and/or in relation to the diet-related NCD burden is sufficient to reduce obesity and diet-related NCDs
**FUND2:** Government funded research is targeted at improving food environments, reducing obesity, NCDs and their related inequalities
**FUND3:** There is a statutory health promotion agency in place that includes an objective to improve population nutrition, with a secure funding stream
**Platforms for Interaction**
**PLATF1:** There are coordination mechanisms across departments and levels of government (national and local) to ensure policy coherence, alignment, and integration of food, obesity and diet-related NCD prevention policies across governments
**PLATF2:** There are formal platforms between government and the commercial food sector to implement healthy food policies
**PLATF3:** There are formal platforms for regular interactions between government and civil society on food policies and other strategies to improve population nutrition
**Health in all Policies**
**HIAP1:** There are processes in place to ensure that development of all government policies relating to food are sensitive to nutrition, public health, and reducing health inequalities in vulnerable populations

Adapted from Swinburn et al. (Swinburn et al., 2013)

#### 2.1.2 Step II: Compilation of evidence and verification by the Ghanaian government

Evidence of government action in each of the policy and infrastructure support domains, and across all 44 indicators, was systematically identified and collected *via* a six-step process (see Box 1)

Box 1 **Ghana Food EPI-specific steps for identifying and reporting evidence.**

Step One - Use the stakeholder mapping to identify key public/government organisations involved in the various Food-EPI policy and infrastructure support domains; also identify key organisational websites.

Step Two - Where organisational websites are identified, trawl each website to identify evidence on relevant policies and/or infrastructure support – capturing these using a Google form and coding the evidence to the relevant Food-EPI domains/indicators.

Step Three - Where no organisational websites are identified and/or once websites have been mined for information, follow up with key identified organisations to discuss what evidence exists in relation to the different policy and support domains.

Step Four – When key policies and/or initiatives have been identified, conduct additional but focused searches of academic databases using key terms associated with any identified policies/initiatives.

Step Five – Submit Official Information Requests to relevant government ministries, departments, and agencies to retrieve information on budgets or other aspects on policies, actions or infrastructure support that may not be publicly available.

Step Six – Follow up with particular stakeholders to discuss the emerging evidence in order to initially validate the emerging evidence and/or to collect further evidence/fill any identified gaps.

As a result, a systematic ‘policy scan’ of publicly available documents evidencing the extent of implementation of the 44 food environment policy and infrastructure support indicators was completed. Evidence of government policy action from the period 2007–2017 was eligible (with one exception: The Ghana Food and Drugs Act. 199, dated 1992, is an active Legislation, although a significant amount of its content has been introduced into the National Public Health Law of 2012. Both were used). Government websites, websites of other institutions (e.g. FAO, WHO, UNICEF) and academic databases (for peer-reviewed journal articles) were systematically searched for evidence of action. Requests for information were also submitted to relevant government authorities.

A data charting spreadsheet facilitated the harvesting of data from the resources. Potential sources of evidence were captured systematically in a shared team database. All identified resources were screened in two stages - first to confirm relevance to any of the 13 Food-EPI domains, and second, a more thorough review to identify suitability for inclusion and data extraction (ie. extraction of detailed relevant content about policy action). Evidence of action was extracted systematically into a shared team database. This process facilitated the generation of a draft ‘evidence paper’ (Laar et al., 2018), which was shared with relevant government stakeholders (e.g. Ministry of Health, Food and Drugs Authority, National NCD Programme) for validation. Although, no additional resources were identified by government stakeholders during validation, the National NCD Control Programme of the Ghana Health Service suggested some content edits. In the final evidence paper, evidence of action taken by the Government of Ghana to create healthier food environments was presented for each good practice indicator alongside examples of international best practice (see Annex 1).

#### 2.1.3 Step III: Assessment of implementation gaps by local expert panel

In total, 41 experts from academia (n = 10), government sectors (n = 15), and non-governmental organizations/civil society (n = 16) were invited to participate in a full-day workshop to rate the extent of government food environment policy and infrastructure support action in relation to: 1) international best practices and 2) an in-country policy cycle. Two weeks prior to the rating workshop, the Food-EPI evidence paper, along with rating instructions, was disseminated to all 41 experts. Written informed consent to participate was obtained, as well as any declaration of conflicts of interest prior to the rating process. During the workshop, experts rated government action in relation to all 13 of the policy and infrastructure support domains and 43 related good practice indicators (Table 1) – one indicator on food hygiene was not rated because international best practice examples were absent. The level of government action against an in-country policy cycle was categorised as: *‘initiation', ‘in development’, ‘implementation’* or *‘evaluation’.* The level of action against international best practice was rated and categorised into *‘high’, ‘medium’, ‘low’* or *‘very little, if any’* (rating instructions are included as Annex 2). The Expert Panel’s ratings were informed by the evidence paper (Laar et al., 2018). To ensure anonymity, the rating for each indicator was conducted online using an electronic tool designed using *Ona Collect.*

We included a constraint in the rating process that required all the expert panel to propose an action, or actions following each rating task. Raters had the option to input “no action required”. The research team reviewed the actions proposed in order to remove duplicates and develop a consolidated list of proposed actions. The consolidated version of proposed actions (n = 20 of the policy actions, and n = 54 of the infrastructure-support actions) in a plenary was projected onto a large screen and the panel discussed both the content and wording of the actions. Following discussions and shortlisting, final proposed actions for policy (n = 13) and infrastructure support (n = 14) were tabulated for prioritization (Annex 3).

#### 2.1.4 Step IV: Prioritization of actions and dissemination of findings

Following the rating workshop, the 19 experts who had participated in the exercise, and 6 others who had been involved in the Food-EPI process but who had missed the workshop, were invited to independently prioritize the proposed actions using online software. The policy and infrastructure support actions were prioritized by each expert separately, though guided by two common criteria: 1)importance (relative need, impact, effects on equity, and any other positive and negative effects of the action) and 2) achievability (relative feasibility, acceptability, affordability, and efficiency) (Annex 3). Following the prioritization process, a final report of the Food-EPI findings, and other findings of the DFC Project were presented to the DFC stakeholders at a deliberative workshop organized in January 2019.

### 2.2 Statistical analysis

Descriptive statistics were generated using Microsoft Excel®. The mean rating for each good practice indicator was used to determine an overall percentage level of implementation. Mean ratings were then categorised into the following levels of implementation based on cut-off points: >75% =‘High’; 51 to 75% =‘Medium’; 26 to 50% =‘Low’; ≤25% =‘Very little implementation–if any’. Differences in ratings based on experts’ background, i.e. ‘government’ versus ‘non-government’ were tested. Agreement (inter-rater reliability) among these two groups of participants was assessed using the Gwet AC2 coefficient with the AgreeStat software (Agreestat 2013.1, Advanced Analytics, Gaithersburg, United States of America). As specified in the Food-EPI protocol (Swinburn et al., 2013) this assessment is a measure of reliability of the Food-EPI assessment tool, especially for comparisons over time.

## 3 Results

### 3.1 Characteristics of evidence on government action on food enviroments in Ghana

The policy scan identified 41 potentially relevant documents detailing evidence of government action on food environments. Documents screening and mapping of content in relation to the 13 Food EPI domains and good practice indicators resulted in the inclusion of 20 documents (see Fig. 2a, Fig. 2b).

**Fig. 2a:**
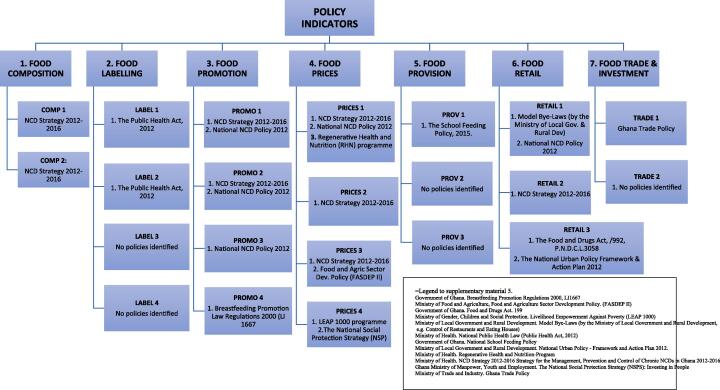
Identified evidence mapped to the *policy-related indicators* of the Food-EPI Domains

**Fig. 2b:**
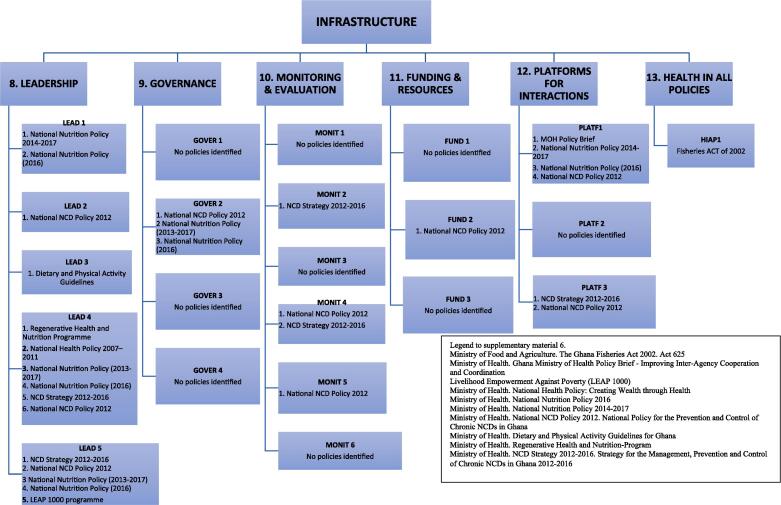
Identified evidence mapped to the *infrastructure support-related indicators* of the Food-EPI Domains

### 3.2 Characteristics of local expert panel

Of 41 multi-sector experts invited to the rating workshop, 12 were non-responsive, 5 sent apologies. On the day of the workshop, poor weather made travel difficult, resulting in 5 cancellations. A total of 19 experts participated in the rating. There were slightly more males (n = 11) than females (n = 8) and an equal distribution of government (n = 9)/non-government sectors (n = 10). Experts had an ‘average’ work experience in their current role of 12 years. All of the 19 experts who had participated in the rating exercise, and 6 others who had been involved in the Food-EPI process but who had missed the workshop, participated in the prioritization exercise.

### 3.3 Implementation of food environment policies and infrastructure support compared with international best practice

Three-quarters of all good practice indicators were assigned a ‘low’ rating in relation to international best practice, including: current government action on food prices (e.g. taxes and subsidies), food retail, food provision and unhealthy food promotion to children, actions relating to political leadership to ensure strong support to create healthy food environments; regular monitoring of NR-NCD risk factors and prevalence and monitoring of food environment. Limited government-funded research on food environments and NCD control was identified as a particular gap and assigned a ‘very little’ rating. However, government effort towards restricting the marketing of breastmilk substitutes was rated ‘very high’ in relation to international best practice exemplars.

**Fig. 3:**
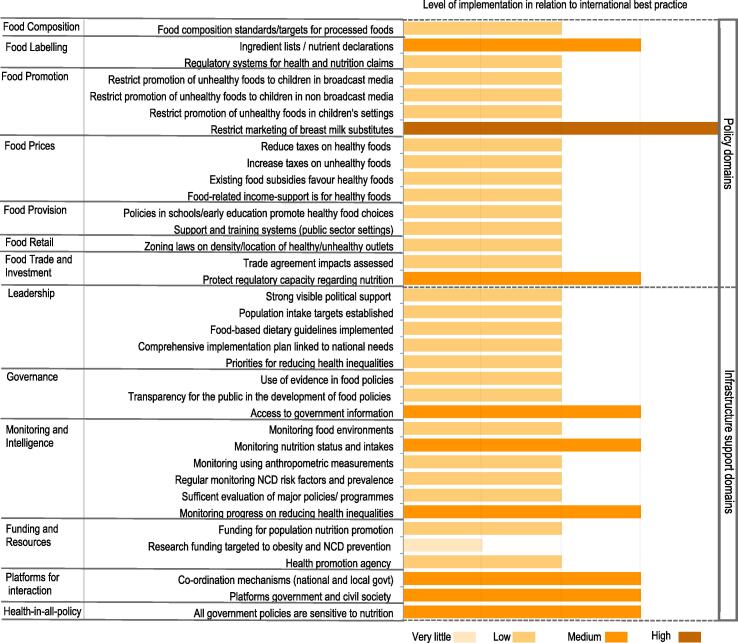
Local expert panel’s ratings of implementation of food environment policies and infrastructure support in comparison with international best practice

Policy action to establish ingredient lists/nutrient declarations was assessed as ‘medium’, as were the efforts of the government to protect regulatory capacities regarding nutrition, in particular through setting standards for maximum fat contents in beef, pork, mutton and poultry. Six infrastructure support good practice indicators, including platforms for interaction across government departments and other sectors (NGOs, private sector, academia), were rated as medium implementation (Fig. 3). No evidence of any government action was documented for 5 policy and 2 infrastructure support areas of good practice, these do not appear in the country scorecard. Inter-rater reliability among all experts was good for ratings made against international benchmarks (Gwet’s AC2 = 0.73 (95% CI 0.66, 0.78)), and no significant difference was found between government and non-government experts.

### 3.4 Extent of implementation of food environment policies and infrastructure support in relation to in-country policy cycle

**Fig. 4:**
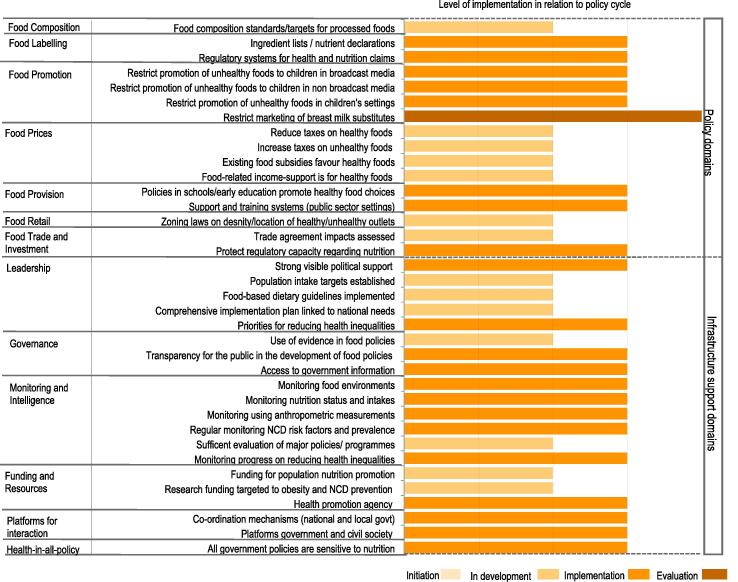
Local expert panel’s ratings of implementation of food environment policies and infrastructure support in relation to stage of local policy action.

The panel also assessed government action in relation to the stage of an in-country policy cycle, i.e. initiation, in development, implementation and evaluation. Government action to restrict the marketing of breast milk substitutes was judged the most advanced (rated as under ‘evaluation’). Twenty-one areas of good practice were rated to be in the ‘implementation’ phase across policy and infrastructure support domains (Fig. 4). No evidence of any government action was documented for 5 policy and 2 infrastructure support areas of good practice, which do not appear in the scorecard (Fig. 4). There has been no action to establish food composition standards for out-of-home meals in food service outlets or a nutrition information system for consumer-oriented labelling on food packaging to enable people to make informed food choices. There was no evidence of action to ensure food service activities in public sector settings (besides schools) promote healthy food choices or to establish platforms for interaction with the commercial food sector or restrict commercial influence on food policy development. Inter-rater reliability among all experts was good (Gwet’s AC2 = 0.79 (95% CI 0.77, 0.82)), and no significant difference was found between government and non-government experts.

### 3.5 Recommended and prioritised policy actions for creating healthier food environments in Ghana

**Fig. 5:**
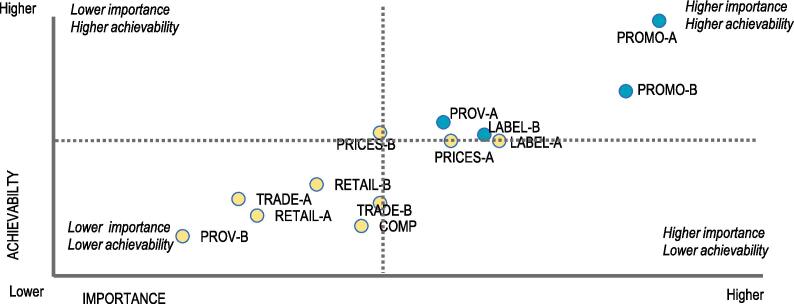
Recommended and prioritized policy support actions for creating healthier food environment in Ghana

A total of 13 policy actions were identified and prioritised taking into account experts’ perceptions of their relative importance and achievability (as mentioned above). Out of these, four actions were prioritised to be of “higher importance” and “higher achievability” (Fig. 5). The top two recommended priorities related to unhealthy food promotion to children: i) legislation to regulate the promotion, sponsorship, advertisement and sale of food and drink with added sugars and other nutrients of concern in children’s settings; and in print and electronic media (PROMO-A); and ii) implementing effective enforcement of this regulation by government (PROMO-B).

### 3.6 Recommended infrastructure support actions for creating healthier food environments

**Fig. 6:**
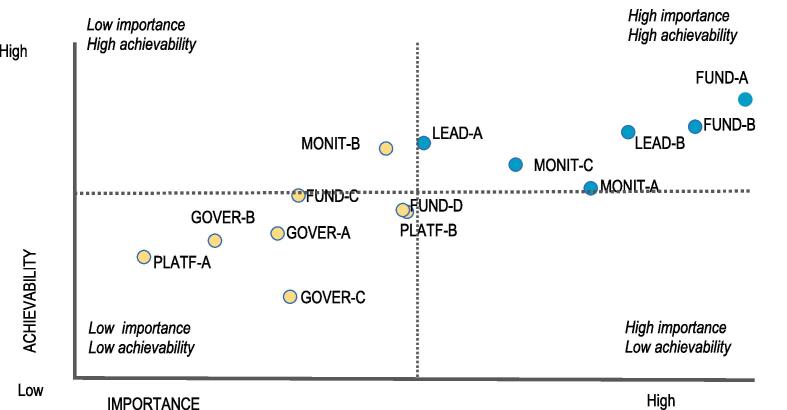
Recommended and prioritized infrastructure support actions for creating healthier food environments in Ghana

A total of 14 infrastructure support actions were identified and prioritised. Out of these, 6 actions were prioritised as of “higher importance” and “higher achievability” (Fig. 6). The highest 2 priorities related to funding and resources; specifically, ensuring sufficient funding for i) addressing nutrition issues and ii) nutrition-relevant research. The other recommended infrastructure support actions related to leadership (the establishment of national food-based dietary guidelines); monitoring and evaluation, particularly, the development of a food composition database; monitoring of the food environment; and the establishment of guidelines on salt intake in line with WHO recommendations.

## 4 Discussion

Ghana is currently experiencing a rise in obesity and other NR-NCDs (Ofori-Asenso et al., 2016, Ghana Statistical Service, 2015, Ghana Statistical Service, 1993). We appraised the Ghanaian government’s response to this changing public health landscape by assessing efforts to improve the healthiness of food environment, thereby controlling obesity and NR-NCDs. This is one of the first such NCD policy appraisals deploying the INFORMAS’ Food-EPI tool in a LMIC setting. The study identified many gaps in the implementation of food environment-policies compared to international best practice, and recommends clear actions prioritized by local experts to improve the Ghanaian food environment.

Although important gaps in policy implementation and accompanying infrastructure support were identified, opportunities exist to improve local food environments and respond to NR-NCDs. Over the past decade, the Ghanaian Government has demonstrated political commitment to the prevention and control of NCDs by developing the National Policy for the prevention and control of NCDs in Ghana (Ministry of Health [MOH-Ghana], 2012) and an accompanying Strategy. These national policies outline actions to meet targets set in globally agreed commitments overseen by the WHO (United Nations General Assembly. UN General Assembly 66th Session Political declaration of the High-level Meeting of the General Assembly on the Prevention and Control of Non-communicable Diseases. A/66/L.1. Sept 16, 2011, World Health Organization. Global strategy on diet, physical activity and health: a framework to monitor and evaluate implementation., 2006, World Health Organization, 2013). To specify, the current declarations by government to have systems that ensure that, where possible, processed foods minimize the energy density and the nutrients of concern (salt, fat, saturated fat, trans fat, added sugar) (Ministry of Health [MOH-Ghana, 2012) is an opportunity. In Ghana, there is a legal framework in place to regulate prohibited claims or potentially misleading claims being made about food (Republic of Ghana, 2012). Other opportunities exist to improve the Ghanaian food environment through implementation of food promotion regulations, and fiscal policies. The Ghana Ministry of Health has expressed desire to protect children from unhealthy food advertisements. The National NCD Policy and Strategy contain intents to formulate legislations prohibiting the advertising of unhealthy foods and non-alcoholic beverages particularly to children. The Ministry of Health, and its agencies – the Ghana Health Services, Food and Drugs Authority, and dedicated programmes – the National NCD Control Programme – are mandated to lead this.

Despite this political will, Ghana’s progress in achieving the global targets referred to above has been slow. The gaps relating to absence of policies, and where policies exist, lack of implementation to promote healthy food environment are partly due to Ghana’s health service delivery system challenges and priorities. Laar et al (Laar et al., 2019) discuss the various challenges associated with managing NCDs in Ghana. Like many LMICs, Ghana’s fragile health system is dealing with multiple burdens of disease and other health system challenges. Having traditionally focused on addressing communicable diseases and maternal and child health, efforts by the Ghana health delivery system to address NCDs are not only nascent, but scant, and not effectively coordinated (Laar et al., 2019). Accordingly, the Ghana expert panel was unanimous in their call for government to design and implement policies, as well as the required infrastructure support for healthier food environments. The panel prioritized legislation to control promotion/advertising of unhealthy food and beverages in the media, in and around schools; compulsory healthy meal planning for school caterers; and support for nutrition advocacy and labelling as high importance and feasibility/achievability. However, they prioritised a mandatory front-of-pack labelling scheme and fiscal measures such as subsidising cost of healthy foods as of high importance but less feasible. We draw on existing literature (Turner et al., 2018) and our collective appreciation of the LMIC food environment to clarify the labelling recommendations of the Ghana panel. Turner et al. (2018) identify two main types of food sources for any food environment – market-based food sources and non-market sources (such as own-production, wild harvested foods, and transfers – including gifts). Non-market sources, which play a key role in Ghanaian food environments, as in other LMICs, pose real challenges for labelling schemes. The market-based food sources in LMICs are equally complex. Ranging from informal street vendors and wet markets, to more formalized shops, specialty stores, cooperatives, ration shops, restaurants, as well as national supermarkets to multi-national supermarket chains. Without rigorous enforcement, it is easy for the informal sector to evade labelling interventions. On fiscal measures, recent challenges encountered by South Africa and Morocco may have influenced the recommendations of the Ghana panel. Currently, in Africa, only South Africa has successfully introduced such fiscal measures – sugar-sweetened beverage (SSB) taxes (Du et al., 2018). Morocco was forced to repeal its tax in November 2018 before it could be implemented in January 2019 due to commercial pressure from the agri-food industry (Bazza, 2019). In South Africa, it took the concerted effort, resources, and courage of civil society, academia, and government to defeat the fervent resistance to the tax by food companies. Public health advocates need courage. As Clive Staples Lewis put it, ‘‘Courage is not simply one of the virtues, but the form of every virtue at the testing point.’’ We argue that a public health advocate without courage, cannot promote health.

Of note, globally, between 2014 and 2018, the Food-EPI tool has been successfully implemented in 11 countries: six in HICs, five UICs, none in LMICs or LICs (Vandevijvere et al., 2019). In comparison with regulatory approaches, standards, and fiscal measures in other jurisdictions, the current findings suggest that the effort of the Ghanaian government at creating healthier food environments is suboptimal (only 1/43 indicators was rated high). Higher implementation has been reported for New Zealand (7/47) (Vandevijvere et al., 2017), and Thailand (5/30) (Phulkerd et al., 2017); but not Malaysia (0/47) (Ng et al., 2018) or England (0/48) (Watson et al., 2018), and South Africa (nearly 90% of the good practice indicators were rated ‘very low if any, or low)’ (Vandevijvere et al., 2019). The current study identified no evidence of the Ghanaian government’s implementation of WHO’s ‘best-buys’ or other recommended interventions for the prevention and control of NCDs (World Health Organization, 2013). The WHO ‘best buys’ related to reducing unhealthy diet include “reducing salt intake through the reformulation of food products to contain less salt and the setting of targets levels for the amount of salt in the food and meals; reducing salt intake through the establishment of supportive environment in public institutions such as hospitals, schools, workplaces and nursing homes, to enable lower sodium options to be provided; reducing salt intake through a behavior change communication and mass media; reduce salt intake through the implementation of front-of-pack labelling”. Effective interventions on the other hand include “eliminating industrial *trans*-fats through the development of legislation to ban their use in the food chain, reducing sugar consumption through effective taxation on sugar-sweetened beverages”.

Like labelling, the implementation of food composition regulatory standards in Ghana was seen as important but there were concerns around how achievable it is. Support was motivated by wider evidence that a food supply system that delivers unhealthy foods (e.g. energy-dense nutrient poor foods, foods with high levels of added sugar, sodium, saturated fats and trans fats), contributes to the rise in obesity and other NR-NCDs (United Nations, 2018]. 2018., Neal et al., 2013, Vandevijvere et al., 2015). Food composition tables are required for Ghana to implement several policies. Firstly to set food industry controls on the level of saturated fatty acids, trans fatty acids, free sugars and salt in processed food products, as recommended by the WHO (World Health Organization, 2006), or to establish Food-Based Dietary Guidelines (FBDGs), which facilitates healthy eating at the population level. The introduction of marketing controls to children in Ghana and food labelling will require good quality data on which foods or beverages need to be ‘controlled’, which would necessitate food composition tables, food-based dietary guidelines, and subsequent nutrient profiling of foods and beverages. This call to action is particularly urgent given that the Ghana food composition tables have not been updated since 1975 (Ankrah and Eyeson, 1975). The Food Research Institute of the Council for Scientific and Industrial Research (CSIR) has the mandate to work with relevant stakeholders to deliver this.

Informed by the impact of food promotion on dietary behaviours and therefore on public health, the need to tackle food promotion that targets children has been averred (Kelly and King, 2015). Several reports indicate that the heavy marketing of fast food and energy-dense nutrient-poor foods and beverages is a ‘probable’ causal factor in weight gain and obesity in children (Cairns et al., 2013, Kelly and King, 2015). The World Health Assembly (WHA), through resolution WHA63.14, provides a set of recommendations to guide efforts by Member States in designing policies, or strengthening existing policies, on food marketing to children, in order to reduce their impact health. Although Ghana’s NCD policy and accompanying strategy declare the government’s desire to formulate legislation prohibiting promotion of unhealthy foods to children, unhealthy foods remain highly advertised and sold (Mark Green et al., 2018). The Ministry of Health and its agencies (especially the Food and Drugs Authority), local government agencies, as well as authorities of child-serving settings need to be supported and prodded to implement these guidelines.

We were intrigued by the expert panel’s recommendation that “the Government should prioritize food transfer over cash transfer when providing support to vulnerable individuals/households”. We noted that the question of whether food or cash is better as a social protection intervention is still undergoing debate. Our work (Laar et al., 2017) reviewed literature in an attempt to provide an answer to the Ghana government on this question. The available literature to that date showed that cash seems to be most appropriate “when markets work adequately; when food is available and affordable; when prices are relatively stable and predictable; when there is a basic financial infrastructure; when there is appropriate capacities in delivery and monitoring; when there are adequate contingency plans; and after harvest”, and least appropriate when the above conditions do not hold or to be less appropriate in situations of limited levels of education. Thus, the decision as to what type of transfer to apply (cash, food or a combination of the two) should necessarily be taken in relation to programme objectives, or in the absence of such conclusive evidence, the answer might as well lie with integrated programmes.

As discussed herein, implementation of fiscal, regulatory, and legislative levers aimed at improving the food environment also recognizes the menu of policies recommended by WHO (World Health Organization, 2013), and confronts commercial drivers of NCDs head-on. The Food-EPI exercise shows that opportunities exist to introduce such interventions in Ghana and address obesity and NR-NCDs. Commitments and declarations of interests to do this, as discussed, exist in Ghanaian government policies. Second, the policy, and infrastructure support actions prioritized as higher-importance and higher-feasibility for the government of Ghana to implement towards preventing obesity and NR-NCDs align with local policies (Ministry of Health [MOH-Ghana], 2012), and global resolutions (WHA63-14) and declarations (United Nations General Assembly, 2011).

The strength of the present paper is inherent in the Food-EPI process/methodology. The process is characterized by broad and active engagement with relevant stakeholders across government, and non-government sectors, which allows for the monitoring of policy implementation over time. Nevertheless, there are potential limitations worth discussing. Limitations described elsewhere on evidence compilation (Vandevijvere and Swinburn, 2014) may apply in the African context. To anticipate this, we deployed counter measures, such as adopting a multi-layered and flexible approach to evidence compilation. Although inaccessibility of government documents is reported elsewhere as a limitation, detailed national budgets in Ghana are publicly accessible online. Another potential limitation is the number of experts (n = 19) who participated in the rating/prioritisation process. However, their diverse scope of expertise and experience (12 years on average) make representation adequate. More importantly, as the Food-EPI exercise is a process, and not an event, the rating/prioritisation exercise was one of many steps. The process convened key stakeholders from academia, civil society organizations, non-governmental organizations, government ministries, departments, and agencies participated in the various Food-EPI engagements prior to the rating workshop. Phulkerd and colleagues have hinted that civic interest may be a major factor influencing experts’ policy ratings (Phulkerd et al., 2017). In their study, state actors were more positive about the government’s performance of policy implementation than non-state actors for the majority of indicators (Phulkerd et al., 2017). Although such differences may be influenced by motivational biases of state actors, it is worth noting that, independent experts may not be exposed to ‘on the ground’ implementation of the relevant policies, and so may not have had complete information regarding the level of implementation.

It is also worthy of note that the Food-EPI tool and process thus far have not adequately addressed ecological sustainability or malnutrition in all its forms. The main focus of the Food-EPI process, as originally developed, is creating healthier food environments to reduce obesity and diet-related NCDs, although several of the actions to promote healthy food environments proposed in the tool could potentially contribute to preventing other forms of malnutrition (Hawkes et al., 2019, Pradeilles et al., 2019). The five policy actions listed by the WHO (World Health Organization, 2017) as potential double duty actions are already incorporated to some degree in the Food-EPI tool (e.g. promotion of breastfeeding, and appropriate complementary feeding in infants; regulations on marketing; maternal nutrition and antenatal care programmes; school food programmes and policies). Of note, the Ghana Food-EPI exercise introduced an indicator relating to supporting, promoting, and protecting exclusive breastfeeding. The vast majority of the infrastructure-support actions are relevant to addressing multiple forms of malnutrition. As many countries are facing multiple burden of malnutrition, it is pertinent to further adapt the tool so that it is responsive to malnutrition in all its forms, particularly, in LMIC settings, and to prioritize double and triple duty actions – in line with the recommendations from two recent Lancet Commissions – “The global syndemic of obesity, undernutrition, and climate change” (Swinburn et al., 2019) “The EAT–Lancet Commission on healthy diets from sustainable food systems (Willett et al., 2019), and the Lancet Series on Double Burden of Malnutrition (Hawkes et al., 2019).

## 5 Policy implications

As discussed, the public health response to the problem of obesity/NR-NCDs is outpaced by their rate of increase. The cost of policy inaction or inertia will result in incalculable losses to public health. We have discussed prioritised policy actions/public health interventions that when implemented by the Ghanaian government would prevent these losses. Taken together, the findings have policy, practice, and public health advocacy implications for Ghana and other LMIC settings. For Ghana, this serves as a baseline benchmark for the government, surveillance of current and future policies will be possible if researchers, and civil society work together for policy implementation to promote healthier diets to prevent NR-NCDs. The paper recognises that given the scope and complexity of NR-NCDs, prevention strategies and policies across multiple levels are required. For instance, policy actions focused on impacting relative availability of healthy versus unhealthy foods (food promotion and fiscal policies) could reduce problematic over‐consumption of energy-dense nutrient-poor foods. These prioritised actions include the introduction of legislation to regulate the promotion/sponsorship/advertisement and sale of unhealthy food and drinks in school environments and in the media, adopting a mandatory food labelling scheme and implementing subsidies to increase the affordability of healthy foods and taxes on unhealthy foods to make them unattractive would require new legislations. Improving the funding environment for nutrition in general is an action to improve infrastructural support towards creating healthier food environments, preventing obesity and diet-related NCDs. We have highlighted the co-dependency of several policy options, which raises the issue of how policy co-ordination will be achieved in Ghana. A multi-sectoral approach will be best accomplished by creating a national alliance to prevent obesity and NR-NCDs supported by high-level leadership in government.

## 6 Conclusions

One of the first such food environment policy appraisals in a LMIC setting, this study identified many gaps in Ghana’s implementation of internationally-recommended policies to promote a healthy food environment. The successful implementation of the process in Ghana, shows that it is possible to deploy the Food-EPI in Africa and other LMIC settings. This has implications on process diffusion to other countries in the sub-region. It is expected that publicizing the outcomes of the current study will contribute towards nurturing African collaborations to combat obesity and NR-NCDs. National stakeholders recommend actions focusing on the macro food environment, which will require legislation. The data support current calls to improve the food environment, but also assert the feasibility of deploying the Food-EPI methodology in Africa.

Contributors

AL: study concept and design, data acquisition, drafting of manuscript; AB: data acquisition, data analysis and preparation of figures; RA: data acquisition; AT: data acquisition, drafting of manuscript; FZ: data acquisition; KB: data acquisition, data analysis and preparation of figures; KM: data acquisition; SV: study concept and design; MH: study concept and design. All authors contributed to revising manuscript and approved the final version.

Ethics

The study received ethical clearance from the Ghana Health Service (GHS) Ethics Review Committee (GHS ERC # 07/09/2016). The University of Sheffield’s Research Ethics Committee a review waiver having recognized that the Ghana Health Service’s Ethics Review Committee has in place sufficiently robust ethics review procedures.

Role of the funding source

The funder of the study played no role in the study design, data collection, analysis or interpretation, nor in the writing of the report. The corresponding author had full access to all the data in the study and had final responsibility for the decision to submit for publication.

Data sharing

The authors agree that the data on which the conclusions of this manuscript rely be deposited in publicly available repositories

## Declaration of Competing Interest

The authors declare that they have no known competing financial interests or personal relationships that could have appeared to influence the work reported in this paper.
