# Supplementary material for: Implementation of healthy food environment policies to prevent nutrition-related non-communicable diseases in Ghana: National experts’ assessment of government action
Source: Food Policy. 2020 May;93:101907. doi: 10.1016/j.foodpol.2020.101907 (PMC7299075; doi:10.1016/j.foodpol.2020.101907)
Supplement: Supplementary data 1 [file mmc1.docx]

**Supplementary material**

**Annex 1 – Food-EPI domains, and selected good practice indicators and associated examples of international best practice**

| **Policy domains** | | | | |
| --- | --- | --- | --- | --- |
| **Domain** | | **Good practice** | | **Examples of international best practice** |
| **Food composition** | | Food composition standards/targets set for processed foods | | **Argentina** Mandatory maximum levels of sodium in various food products. **Denmark** Ban on trans fats |
|  |  | Food composition standards/targets set for out-of-home meals in food service outlets | | **USA** Restaurants not allowed to produce foods that contain partially hydrogenated oils (PHOs). **New Zealand** Industry standards set for deep frying oils. |
| **Food labelling** | | Ingredients lists / nutrient declarations required | | **Canada, USA (and others)** require trans fat labelling on packaged food  **USA** Requirement for added sugar to be included on packaged food labelling |
|  |  | Regulatory systems in place for health and nutrition claims | | **Indonesia** Regulation establish rules on the use of specified nutrient content claims (i.e. level of fat for a low-fat claim). |
|  |  | Front-of pack labelling system | | **Ecuador** Mandatory traffic light labelling indicating healthiness of food products. **Chile** Warning labels for products high in calories, saturated fat, sugar or sodium. |
|  |  | Menu board labelling system | | **South Korea** Chain restaurants (100+ outlets) must display nutrient information on menus (energy, total sugars, protein, saturated fat, sodium). |
| **Food promotion** | | Restrict promotion of unhealthy food to children in broadcast media | | **Chile** No advertising of unhealthy foods directed to children under 14 (or when audience share is greater than 20% children) |
|  |  | Restrict promotion of unhealthy food to children in non-broadcast media | | **Quebec, Canada** Ban on all commercial advertising directed to children (under 13 years) through any medium |
|  |  | Restrict promotion of unhealthy food in children’s settings | | **Spain** Legislation requires that kindergartens and schools are free from all advertising |
|  |  | Restrict marketing of breast milk substitutes | | **Various countries** Legislation / adopted regulations encompass all / nearly all requirements of WHA International Code on this topic. |
| **Food prices** | | Reduce taxes on healthy foods | | **Fiji** Removed excise duty on imported fruits, vegetables and legumes. |
|  |  | Increase taxes on unhealthy foods | | **Mexico** 10% tax on sugary-drinks, 8% tax on unhealthy snack foods. **Hungary** Public health tax on sugary-drinks / various unhealthy foods. |
|  |  | Existing food subsidies favour healthy foods | | **Canada** Retail-based subsidy program in northern isolated communities enables local retailers and registered suppliers to access and lower the cost of perishable healthy foods (e.g. eggs, vegetables) |
|  |  | Food-related income-support is for healthy foods | | **UK** Pregnant women / families with children under 4 who receive certain state benefits receive weekly ‘healthy start’ food vouchers. |
| **Food provision** | | Policies in schools/early education promote healthy food choices | | **Costa Rica** Schools only permitted to sell food meeting set nutritional standards. **UK** Mandatory nutritional standards for all food served in schools-restrictions on high fat/ sugar/salt/processed foods. |
|  |  | Policies in public settings promote healthy food choices | | **New York City, USA** Mandatory nutritional standards for all food purchased/sold by city agencies (hospitals, prisons, aged care, health facilities) |
|  |  | Support and training systems in place in public sector settings | | **Japan** Mandatory oversight / monitoring by dietitian/nutritionist (e.g. menu development) for all government facilities providing >250 meals/day |
| **Food in retail** | | Zoning laws on the density/location of healthy/unhealthy food service outlets | | **South Korea** ‘Green Food Zones’ (<200m) around schools ban the sale of foods (fast food, soda) deemed ‘unhealthy’ by Food and Drug Administration |
|  |  | In-store availability of healthy/unhealthy foods regulated | | **UK** Voluntary agreement with commercial companies to increase availability of fruit/vegetables at convenience stores |
|  |  | Robust food hygiene policies | | NA – new policy area added for Ghana Food-EPI |
| **Food trade and investment** | | Trade agreement impacts assessed | | **European Union** Mandatory environmental impact assessments (potentially including health impacts) for all new trade agreements |
|  |  | Protect regulatory capacity regarding nutrition | | **Ghana** Standards set maximum % fat contents in beef, pork, mutton and poultry. |
| **Infrastructure support domains** | | | | |
| **Domain** | **Sub-area of good practice** | | **Examples of international best practice** | |
| **Leadership** | Strong, visible political support for population nutrition | | **New York City, USA** Mayor (M. Bloomberg) showed strong political leadership in introducing ‘landmark’ food policies, including restrictions on trans-fat and portion size restrictions on sugary-drinks. | |
|  | Population intake targets established | | **Brazil** ‘Strategic Action Plan for Confronting NCDs’ specifies targets for fruit and vegetable consumption, and reductions in average salt intake. | |
|  | Food-based dietary guidelines implemented | | **Brazil** National dietary guidelines address healthy eating from a cultural, ethical and environmental perspective. | |
|  | Comprehensive implementation plan linked to national needs/priorities | | **EU** European Food and Nutrition Action Plan 2015-20 outlines clear strategic goals, guiding principles, objectives, priorities and tools. | |
|  | Priorities for reducing inequalities related to nutrition | | **New Zealand** Ministry of Health upholds contracts with NGOs/other institutions to prioritise Maori health and Maori specific needs in service delivery, service development and planning | |
| **Governance** | Restricting commercial influence on policy development | | **Australia** Public Services Commission Values and Code of Conduct includes a number of relevant sections (e.g. conflicts of interest, lobbying) | |
|  | Use of evidence in policies related to population nutrition | | **Australia** National Health and Medical Research Council requirements to develop evidence-based guidelines | |
|  | Transparency in the development of food policies | | **Australia** Open access principles across governments, FSANZ processes for extensive stakeholder engagement in the development of new standards | |
|  | Publicly available nutrition and policy information | | **Various countries** ‘Freedom of Information’ legislation provides certain rights of public to access documents of government departments/agencies. | |
| **Monitoring and Evaluation** | Monitoring food environments | | **New Zealand** Database of nutrient information for different foods, monitoring of school food environments nationwide | |
|  | Monitoring population nutrition status and intakes | | **USA** National annual survey provides detailed national information on health status, disease history and nutritional intake of adults and children | |
|  | Monitoring population body weight | | **UK** National Child Measurement Program for children’s BMI, assessing children ages 4-6 and 10-11 | |
|  | Monitoring of NCD risk factors | | **OECD countries** Most have robust prevalence, incidence and mortality data for the main diet-related NCDs and NCD risk factors | |
|  | Evaluation of major programs and policies | | **USA** The National Institutes for Health has dedicated funding for evaluating new policies/programs expected to influence obesity- related behaviours | |
|  | Monitoring of inequalities in relation to nutrition | | **New Zealand** All annual Ministry of Health surveys estimate by subpopulations | |
| **Funding and Resources** | Funding for nutrition as a proportion of total health spending | | **Thailand** Expenditure report from 2012 showed the government had increased spending on nutrition (excluding food, hygiene control). | |
|  | Research funding for obesity and other NCDs | | **Thailand** National Research Council funded more projects on obesity and diet-related chronic diseases between 2013 to 2014. | |
|  | Statutory health promotion agency with sustainable financing | | **Australia** The Victorian Health Promotion Foundation is an autonomous government agency established as a dedicated health promotion agency. | |
| **Platforms for interaction** | Coordination mechanisms across departments / levels of government | | **Malta** Inter-Ministerial Advisory Council on Healthy Lifestyles (cross-sectoral group) advises the Minister on Health with a life-course approach to nutrition. | |
|  | Platforms for government and commercial food sector interaction | | **UK** ‘Responsibility Deal’ was an initiative to bring food companies and non-government groups together to address NCDs. | |
|  | Platforms for government and civil society interaction | | **Brazil** The National Council of Food and Nutrition Security (CONSEA) is made up of civil society and government representatives that advises the President’s office on matters involving food and nutrition security. | |
| **Health in all policies** | All government policies sensitive to nutrition and inequalities | | **Slovenia** Undertook a Health Impact Assessment in relation to agricultural policy at national level. | |

**Annex 2 – Rating instructions**

Sir/Madam,

You are invited to participate in the 2018 Ghana Food-EPI expert panel. You will be required to rate the **current** level of implementation of each of the 44 good practice indicators by the Government of Ghana, – first in terms of extent of progress against international **against international best practices,** and second, based on **progress within a policy cycle framework.** Each will be scored on a five-point Likert scale using a paperless survey questionnaire.

Meaning of Likert Scale for rating exercise one:

**Rating of extent of progress against international policy benchmarks.**

| 1 (<20% implementation ie. very low to no progress in relation to best practice) |
| --- |
| 2 (21-40% implementation ie. low to medium progress in relation to best practice) |
| 3 (41-60% implementation ie. good progress in relation to best practice) |
| 4 (61-80% implementation ie. very good to excellent progress in relation to best practice) |
| 5 (81-100% implementation ie. excellent progress in relation to best practice) |
| 6 Cannot rate |

**If cannot rate, why……………………………………………………….?**

Meaning of Likert Scale for rating exercise two:

**Rating of extent of implementation in Ghana in terms of progress within a development cycle framework.** Please rate each indicator according to where progress and implementation best fit within the following process cycle stages:

1. Agenda setting/initiation phase
2. Development phase
3. Implementation phase
4. Evaluation phase
5. Cannot rate

**If cannot rate, why………………………………………………………….?**

NOTE: Regarding the ‘cannot rate’ option: please only use this if really needed and provide comments in the comment box on why you cannot rate for a particular good practice indicator.

NOTE: The ratings require expert judgement, taking multiple considerations into account. For instance, quality of government policies/actions compared to international best practice (as in exercise 1); extent of implementation of government policies/actions considering all aspects of the ‘policy cycle’ (as in exercise 2).

NOTE: You will need to consider the intentions and plans of the Government of Ghana funding, funding for implementation of actions undertaken by NGOs and establishment of working or advisory groups, etc., in addition to the policies and actions that have been implemented.

**This Evidence Pack** gives you the full details of the current evidence of implementation by the Ghanaian Government for each good practice indicator and includes international best practice examples (benchmarks) for each good practice indicator to **support you** in the rating process.

**It is important to read the evidence of implementation and international benchmarks before putting in your rating for each good practice indicator.**

**Annex 3 – Instructions for prioritizing actions for Government of Ghana and other stakeholders**

Dear Expert/Stakeholder,

As a relevant stakeholder and/or member of the Food EPI Expert Panel, we invite you to prioritise a set of recommended actions to improve the food environment in Ghana, that local experts/stakeholders generated at the recent Food EPI Workshop (held on 19th September 2018 in Accra).

You will be asked to prioritise policy-speciﬁc options and infrastructure support actions.

Please read the instructions on the next page, then start the prioritisation process. The entire process should take approximately 25-30 minutes in total.

**INSTRUCTIONS**

**STEP ONE**: Familiarise yourself with the actions that are included in the prioritisation process.

**STEP TWO**: Then prioritise each of the actions in rank order using two criteria: 1. IMPORTANCE and 2. ACHIEVABILITY. The policy-speciﬁc actions need to be ranked in order from 1 – 13 for importance and for achievability (where **1 = most**

**important/most achievable and 13 = least important/least achievable)**.

The infrastructure support actions need to be ranked in order from 1- 14 for importance and for achievability (where **1 = most important/most achievable and 14 = least important/least achievable**)

EXPLANATION OF THE CRITERIA:

1. **IMPORTANCE - Please take the following into account:**

•Need: The size of the implementation gap.

- Equity: Progressive/regressive eﬀects on reducing food/diet-related health inequalities
- Other positive eﬀects: For example, on protecting rights of children and consumer
- Other negative eﬀects: For example, regressive eﬀects on household income, infringement on personal liberties
- Impact: The eﬀectiveness of the action on improving food environments and diets (including reach and eﬀect size).

1. **ACHIEVABILITY - Please take the following into account:**

- Feasibility: How easy or diﬃcult the action is to implement
- Acceptability: The level of support from key stakeholders, including government, public health and industry
- Aﬀordability: The cost of implementing the action
- Eﬃciency: The cost-eﬀectiveness of the action

**NOTE:**

The prioritisation results will indicate which actions the expert group think are most important and the most achievable for Government of Ghana to implement.

Unhealthy foods refer to Processed foods or non-alcoholic beverages high in saturated fats, trans fats, added sugars and/or salt.

'Foods' refer to 'foods and non-alcoholic beverages'. Alcohol is excluded from the Food EPI framework.

1. **Please indicate the best representation of your current position: (you may put X by one of the radio buttons below)**

Government oﬃcial

Non-governmental/civil society representative

Academic/researcher

Private sector representative

POLICY-SPECIFIC ACTIONS

Please prioritise each of the policy-speciﬁc actions in rank order from 1-13 using two criteria:

- 1. **IMPORTANCE (where 1 = most important and 13 = least important). and**
  2. **ACHIEVABILITY (where 1 = most achievable and 13 = least achievable)**

**Please make sure that no two actions are given the same rank. If the need arises for the rank of an action to be revised, you are allowed to do that.**

| **ACTIONS** | **IMPORTANCE** | **ACHIEVABILITY** |
| --- | --- | --- |
| The Government through the relevant agency, (eg. Food and Drugs Authority; Ghana Standards Authority) should set food composition standards for out-of-home meals. |  |  |
| The Government should adopt a mandatory labelling scheme that ensures that foods manufactured for both local and international markets are appropriately labelled. (e.g. develop mandatory front-of-pack labelling such as the traﬃc light labelling scheme. |  |  |
| The Government should support nutrition advocates (e.g. with ﬁnancial support, knowledge and research development, capacity planning). |  |  |
| The Government should institute a requirement for all restaurants to have appropriately qualiﬁed nutritionists and dietitians on staﬀ |  |  |
| The Government should pass a legislation to regulate the promotion, sponsorship, advertisement and sale of food and drink with added sugars, and other nutrients of concern (saturated fatty acids/trans fats, salt) in the school environment and other child-laden settings, enforceable with ﬁnes. |  |  |
| The Government should enforce legislation to regulate the promotion, sponsorship, advertisement and sale of food and drink with added sugar, and other nutrients of concern (saturated fatty acids/trans fats, salt) in print and electronic media, enforceable with ﬁnes. |  |  |
| The government should implement subsidies to increase the aﬀordability of healthy foods. |  |  |
| The government should implement taxes on unhealthy foods that will raise their price. |  |  |
| The Government should implement a requirement for caterers involved in the School Feeding Programme to pass a training course on healthy meal planning. |  |  |
| The Government should prioritize food transfer over cash transfer when providing support to vulnerable individuals/households. |  |  |
| The Government should ensure that local authorities are equipped with the requisite resources to monitor unhealthy foods sold in local markets. |  |  |
| The Government should ensure that the impact of trade and investment agreements on food environments, population nutrition and health are assessed and monitored. |  |  |
| The government should develop and implement a strategy to control illegal imports of unhealthy foods. |  |  |

**Please prioritise each of the infrastructure support actions in rank order from 1-14 using two criteria:**

1. **IMPORTANCE (where 1 = most important and 14 = least important). and**
2. **ACHIEVABILITY (where 1 = most achievable and 14 = least achievable)**

**Please make sure that no two actions are given the same rank. If the need arises for the rank of an action to be revised, you are allowed to do that**.

| **ACTIONS** | **IMPORTANCE** | **ACHIEVABILITY** |
| --- | --- | --- |
| The government should ensure that suﬃcient and transparent funding is allocated to nutrition, particularly promotion of healthy eating. |  |  |
| The government should allocate adequate funding for nationally-relevant research on nutrition and NCDs, including obesity and related health and social inequalities. |  |  |
| The government should earmark all revenues collected from tobacco sales to fund health related research, including nutrition. |  |  |
| The government should issue guidelines on recommended daily salt (sodium) guidelines in line with WHO recommendations. |  |  |
| The government should develop and publish food based dietary guidelines. |  |  |
| The government should develop a food composition database. |  |  |
| The government should create a Health Promotion Agency with dedicated funding. |  |  |
| The government should regularly monitor and evaluate indicators of health inequalities with the aim of reducing these and improving the health of vulnerable populations. |  |  |
| The government should establish regular surveillance and monitoring of the food environment, including obesity and overweight in the population across all age groups. |  |  |
| The Government should form a strategic partnership with West African nations to exchange ideas and practices on reporting, surveillance and monitoring and evaluation on population nutrition and NCDs. |  |  |
| The government should ensure that comprehensive nutrition-related information is available and accessible within districts. |  |  |
| The Government should ensure that the Access to Information Bill is passed by Parliament. |  |  |
| The government should develop and implement policies to regulate relationships and inﬂuence of commercial industry on government. |  |  |
| The government should strengthen cross-sectoral platforms for coordination of nutrition and nutrition-related policies and plans. |  |  |

**YOUR THOUGHT ON THE WEIGHTING OF "IMPORTANCE" vs. "ACHIEVABILITY"**

Please let us know if you think Importance and Achievability criteria should be weighted the same or diﬀerently when these scores are combined.

1. **Should these two be weighted 50%: 50%?(you may put X by one of the radio buttons below)**

Yes

No

IF NO, HOW SHOULD THESE TWO BE WEIGHTED?

**Importance**

**Achievability**

**Total**

Thank you for your time. The Ghana Food-EPI team will be in touch with a report/summary of the results in due course. If you have any questions, please contact: Dr. Amos Laar of the University of Ghana *(*[*alaar@ug.edu.gh*](mailto:alaar@ug.edu.gh)*)*
